# Spatial Movement Patterns and Local Co-Occurrence of Nutria Individuals in Association with Habitats Using Geo-Self-Organizing Map (Geo-SOM)

**DOI:** 10.3390/biology10070598

**Published:** 2021-06-28

**Authors:** Do-Hun Lee, Nam Jung, Yong-Hyeok Jang, KyoungEun Lee, Joobaek Lim, Gab-Sue Jang, Jae Woo Lee, Tae-Soo Chon

**Affiliations:** 1 National Institute of Ecology (NIE), Seocheon 33657, Korea; eco0407@nie.re.kr (D.-H.L.); kelee25@nie.re.kr (K.L.); 2 Department of Physics, Inha University, Incheon 22212, Korea; uchpracacia@gmail.com (N.J.); jaewlee@inha.ac.kr (J.W.L.); 3 Ecology and Future Research Institute, Busan 46228, Korea; younghyuck@daum.net (Y.-H.J.); jbleem@hanmail.net (J.L.); 4 Department of Life Sciences, Yeungnam University, Gyeongsan 38541, Korea; sunside@ynu.ac.kr

**Keywords:** small mammal, *Myocastor coypus*, movement behavior, nearest-neighbor distance, sexual difference, machine learning

## Abstract

**Simple Summary:**

Nutrias (*Myocastor coypus*) escaped captivity in the 1990s in Korea; these individuals rapidly established wild populations, causing substantial environmental issues, including biodiversity loss, local habitat disturbance, and agricultural damage. The South Korean government initiated the Nutria Eradication Project in 2014 to control nutrias on a national scale. The aim of this research was to support the eradication efforts by improving our understanding of nutria movements based on biological and environmental factors. In this study, the geo-self-organizing map software was applied to radio-tracking data from individuals, and it was determined that males dominate nutria movement. Movement patterns were seasonal and varied with vegetation types between sexes and within each sex. Tall grassland was mainly associated with interactions between individuals of opposite sexes (possibly related to mating), whereas floating-leaved hydrophytes were related to same-sex interactions (possible feeding grounds). Data from this large-scale monitoring provide initial results for a more targeted and effective eradication program. Further large-scale population dynamics research is needed for a successful eradication program in the future.

**Abstract:**

Nutrias (*Myocastor coypus*) were imported to South Korea for farming in 1985; individuals escaped captivity and established wild populations in natural ecosystems in the late 1990s. Numerous studies have focused on their monitoring and management; however, information on the continuous movement of individuals is not available. In this study, telemetry data from field conditions were used to identify the nearest-neighbor distances of individuals in association with environmental factors, including plant type, land cover, and biological parameters. The minimum nearest-neighbor distances for the different sexes were, overall, according to the minimum distances for the same sex. Local co-occurrences of individuals, either of the same or different sex, were seasonal. Tall grasslands, followed by herbaceous vegetation, were associated with the co-occurrence of different sexes. Conversely, floating-leaved hydrophytes, followed by xeric herbaceous vegetation, were correlated with the co-occurrence of the same sex. Local female–male co-occurrences were negatively associated with male–male co-occurrences but not with female–female co-occurrences, suggesting male dominance in group formations. Movement and co-occurrence information extracted using Geo-self-organizing maps furthers our understanding of population dispersal and helps formulate management strategies for nutria populations.

## 1. Introduction

Industrial development followed by climate change has accelerated the influence of invasive alien species globally, which is critical for population management strategies. Owing to repeated introductions and the establishment of new populations, invasive species continue to disturb ecosystem stability, especially in Korea, since the 1990s [1,2]. Nutrias (*Myocastor coypus*) further exacerbate existing anthropogenic disturbances, including human population aggregations, resource utilization, habitat reduction following urbanization, and environmental pollution, especially under the stressful conditions of global warming [3,4,5,6,7]. International trade and global interactions have also led to the geographical spread of alien species, disturbing native species distribution and faunal stability [8,9,10,11,12].

Nutrias, native to America, are semi-aquatic rodents imported to countries in Europe, Asia, and Africa for meat and fur. However, upon a decrease in demand, farmed nutrias were released into the environment. The rapid settlement of nutria populations has resulted in major issues, including loss of biodiversity, disturbance of local habitats, and damage to agriculture [13,14,15,16,17,18,19,20,21]. Countries that have experienced such problems attempt to control nutria populations with proactive management methods [22,23,24].

Nutrias were initially imported to South Korea in 1985 for farming use. Captive individuals escaped from farms, and by the late 1990s, populations were established in ecosystems [25]. By 2013, nutria populations inhabited 23,384 km^2^ along the Nakdong River in the southeastern area of the Korean peninsula [1,26]. Nutrias prefer wet and temperate climates; populations in the Korean peninsula are mainly limited to southern areas with above-freezing temperatures and wetlands in winter [27,28]. In 2014, the South Korean government initiated the Nutria Eradication Project to eliminate nutrias on a national scale. Since the initiation of this project, active management efforts have occurred, with the capture of 27,000 nutrias in 2018 [1].

Ecological informatics (including machine learning) have been utilized since the 1990s to extract complex community and population data [29,30]. Machine learning techniques, notably MaxEnt, have been used extensively for predicting potential range expansions of species distribution, including those of nutrias, at the population level [31,32]. In this study, self-organizing maps (SOMs) were used at the individual level to train the movement behavior of nutrias. SOMs have been used as an efficient method of information extraction from multidimensional data, to create comprehensive maps based on unsupervised learning [33,34,35]. Since their initial application in patterning community dynamics [36,37], SOMs have been used as a major model for dimension compression, which provides flexibility in data presentation and visualization for ecological research [30,38,39,40,41], environmental sciences [42], and resource management [43]. SOMs have been further used to extract behavior data for the detection of toxic responses [44], diagnosing disease [45], revealing conflict responses [46], and investigating gene–behavior relationships [47,48].

Although associations between species and environmental factors have been determined using SOMs [41], few studies have used spatial data (i.e., sample positions) as the input data. In the present study, Geo-SOM was applied to positional movement data, in association with biological and environmental variables. Geo-SOM was introduced to extract information on covariates of sample units to present community patterns within geographic locations [49,50].

Available telemetry data on nutrias in the spatial domain under field conditions were analyzed, and information on continuous individual movement associated with biological and environmental variables, was extracted using Geo-SOM. It was hypothesized that the movement of individuals and local co-occurrence would be characteristically different according to season, sex, and habitat type. Furthermore, it was speculated that the distances between different sexes would be shorter than those between the same sex, considering that individuals of different sexes would have close relationships, e.g., mating, whereas individuals of the same sex would have hostile relationships, e.g., competition. Particularly, males would have more negative relationships with other males, owing to their aggressive behavior and dominant role in group formation. Consequently, the distances between males would be greater than those between females. Extrapolating male dominant behavior, it was also conjectured that local co-occurrence of the same and different sexes would be more governed by males than by females, being further related to environmental factors, including plant types and land cover states.

The objectives of the current study were (1) to analyze movement parameters, including linear speed during movement; (2) to determine nearest-neighbor distances in association with environmental and biological variables; and (3) to demonstrate the roles of females and males in determining local co-occurrence patterns in field conditions.

## 2. Materials and Methods

### 2.1. Study Area

**Figure 1:**
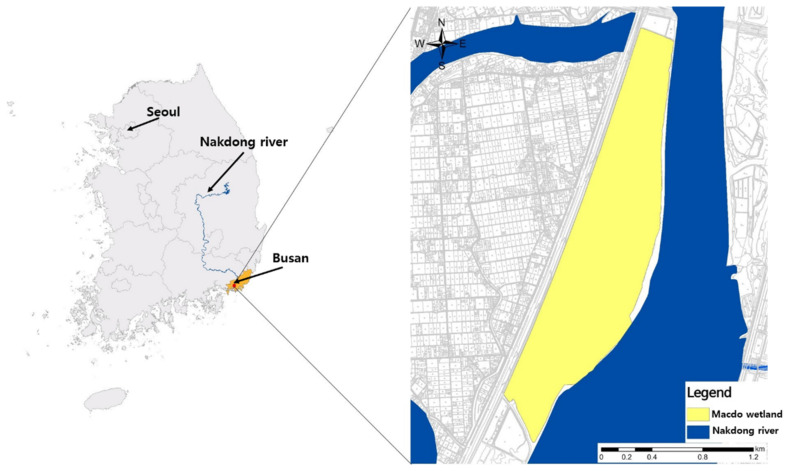
Maps of the Macdo wetland in Korea that were used for surveying the movement of nutria individuals.

The Macdo wetland (126°34′30″–126°39′0″ E, 37°15′0″–37°16′30″ N) is located in Busan Metropolitan City, in the south-east corner of the Korean Peninsula. It occupies 2.58 km^2^, and is rectangular, with a longest side length of 6.90 km (Figure 1). Due to minimal disturbance by humans, the survey area is considered one of the most representative seasonal habitats for migratory birds in Korea. The wetland is also known as a major habitat for nutrias, with reports of habitation since 2009 [1]. The temperature in the Macdo wetland ranges from −3 °C to 2 °C during winter, which is relatively milder than other regions of the Korean Peninsula. In summer, the temperature is fairly uniform, ranging from 25 °C to 26 °C. Monthly average water temperatures in the survey area were obtained from the Water Environment Information System of the National Institute of Environmental Research, from October 2015 to September 2016.

### 2.2. Monitoring of Individual Movement

**Figure 2:**
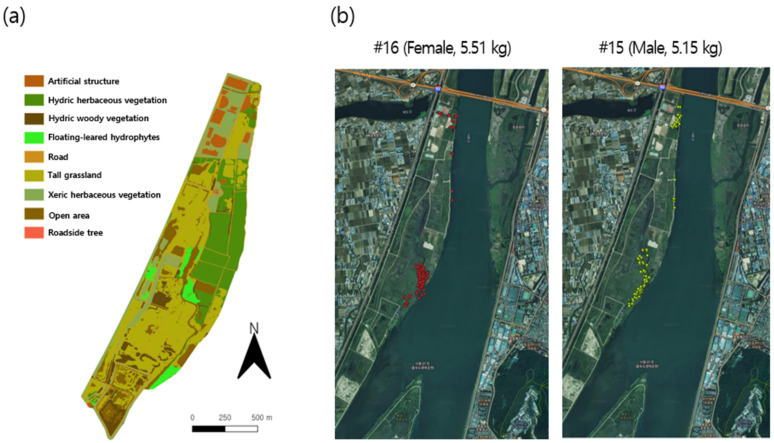
(**a**) Spatial habitat types in the Macdo wetland during the survey period and (**b**) examples of individual movement tracks in the survey area.

The movement of 24 individual (12 females and 12 males) nutrias was monitored in the Macdo wetland from October 2015 to September 2016, using telemetry (Figure 2). The captured population was collected by the Nutrient Eradication Project, initiated by the Ministry of Environment, Republic of Korea.

Twenty-four animals were captured using traps (78 cm × 28 cm × 33 cm; Tomahawk, WI, USA) set in the Macdo wetland, and characteristics of sex, weight, and age were determined under anesthetization with alfaxalone (0.4 mL/kg) (Appendix A). The animals were rested for 48 h, with a radio transmitter (R2030, ATS Inc., Minneapolis, MN, USA) attached to their necks. Seven days after their release to their original locations, radio-tracking of a cohort of individuals was undertaken, with careful consideration of their adaptation to the original habitats. The radio-tracking data were collected at 1–2 h intervals for 3 consecutive days and night each month, using very high frequency (VHF) radio collar antenna (ATS Inc.) and the ground homing method [51]. In total, twelve surveys were conducted during the survey period, with each survey per month (Appendix A).

The location of each animal was confirmed by direct observation after approaching by radio-tracking. Radio-tracking was generally performed using a truck with a VHF antenna (ATS Inc.). A wireless R20 radio receiver (ICOM Inc., Osaka, Japan) was used to receive the location signals, and locations were recorded following the protocols of the GPS receiver (Garmin Inc., Olathe, KS, USA). In particular, the strongest signals were tracked to gain access to the individual and to reduce bias as much as possible. Stress to the individuals was minimized by following the ethical protocols previously established for using radio transmitters on animals [52].

Due to field conditions, and variability in individual behavior, the number of observations varied among individuals. Four individuals (three females and one male) were recorded for more than 150 points, while eight individuals (three females and five males) had more than 100 points (Appendix A). The movement of individual #18 was not recorded due to the loss of the radio transmitter during the survey period. Individuals with a sufficient number of records were included in the analysis (see Section 3.2 and Section 3.3). Figure 2b provides example data for the movement tracking of two nutria individuals in the survey area.

To identify the vegetation distribution in the Macdo wetlands, vegetation surveys were conducted from August to October 2016 based on the Braun–Blanquet’s phytosociological methods [53], and the distribution was classified following Spencer and Bousquin [54]. An actual vegetation map was drawn from the field survey data, with a scale of 1:5000, and analyzed using ArcGIS 10.3.1 (Esri Inc., Redlands, CA, USA). The vegetation map (Figure 2a) indicated vegetation types (such as hydric herbaceous vegetation [HHV] and tall grassland [TG]) and land cover states (such as road [R] and open area [OA]).

### 2.3. Geo-Self-Organizing Map (Geo-SOM)

**Figure 3:**
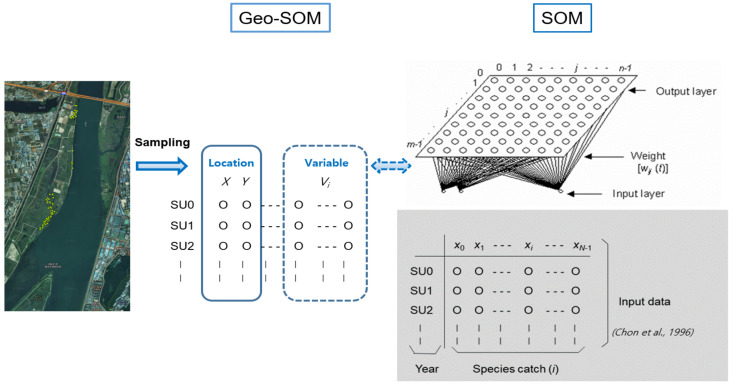
Geo-self-organizing map (Geo-SOM) applied to the patterning of associations among locations, environmental factors, and biological data for movements of nutria individuals.

In the SOM, a linear array of *M*^2^ artificial neurons (i.e., computation nodes), where each neuron is represented as *j*, is arranged into two dimensions to visually interpret the data (right panel, Figure 3. Only variables (e.g., habitat types) without spatial information were used as the input data in the original SOM. It was assumed that the data containing *N* variables and *x_i_* are expressed as the input value for node *i*. In the SOM network, each neuron *j* is connected to node *i* in the input layer. The connectivity is presented as weights, *w_ij_*(*t*), which adaptively change at each iteration of the calculations, *t* (right panel, Figure 3). Initially, the weights were randomly assigned to small values. When the input vector runs through the network, each neuron of the network computes the summed distance *d_j_*(*t*) between the weight and input, as shown below:

$$d_{j}\left(t\right)=\overset{N-1}{\underset{i=0}{\sum }}(x_{i}-w_{ij}{\left(t\right)}^{2})$$

The neuron with the maximum response to a given input vector is selected as the winning node, as its weight vector has the shortest distance to the input vector. The winning node, and possibly its neighboring nodes, are allowed to learn by adjusting the weights in a manner that further reduces the distance between the weight and the input vector, as shown below:

$$w_{ij}\left(t+1\right)=w_{ij}\left(t\right)+\eta \left(t\right)\left(x_{i}-w_{ij}\left(t\right)\right)Z_{j}$$

where *Z_j_* is assigned a value of 1 for the winning (and its neighboring) node(s) through training and is assigned a value of 0 for the remaining nodes. The parameter *η*(*t*) (e.g., 0.1–0.4) denotes the fractional increment of the correction. The radius defining the neighborhood is usually given a larger value early in the training process, and gradually reduces as convergence is reached [36,41,55].

Geo-SOM was designed to specifically reveal the interrelationships among variables, in association with geographic locations. Compared to the original SOM, the location data (e.g., longitudes and latitudes, expressed as ‘*X*’ and ‘*Y*’, respectively) are provided as input (left panel, Figure 3). Geo-SOM training is conducted in two phases: initial location information is provided, and then a geographical vicinity is searched for the best matching unit (BMU). The weight of the spatial information is controlled by the geographic tolerance (*k*; between 0 and 5 in this case), with the lower level indicating a higher weight of the geographic information. Based on preliminary tests, *k* = 3 was selected for training in this study. After selecting the sample units using geographic tolerance, additional training was conducted using new variables (environmental factors and biological data in this study) for the second phase of the Geo-SOM [49,50].

The size of the computation nodes was 9 × 6, which represented the overall variations in the data, following the preliminary studies based on Vesanto et al. [56]. The *k*-means clustering was conducted to determine the number of clusters on the ordination map [57]. The training was performed according to the Geo-SOM source code (www.isegi.unl.pt/labnt/geosom, accessed on 18 June 2021) in the MATLAB^®^ environment compatible with the SOM-toolbox [56], with rough (200 iterations with initial neighbor size and learning rate of 4 [radius] and 0.2, respectively) and fine (20 iterations with initial neighbor size and learning rate of 10 [radius] and 0.1, respectively) training sessions. Multiple comparison tests and *T*-tests [56] were conducted to statistically differentiate clustered variables (e.g., distances between individuals) after training.

## 3. Results

### 3.1. Movement Parameters

**Figure 4:**
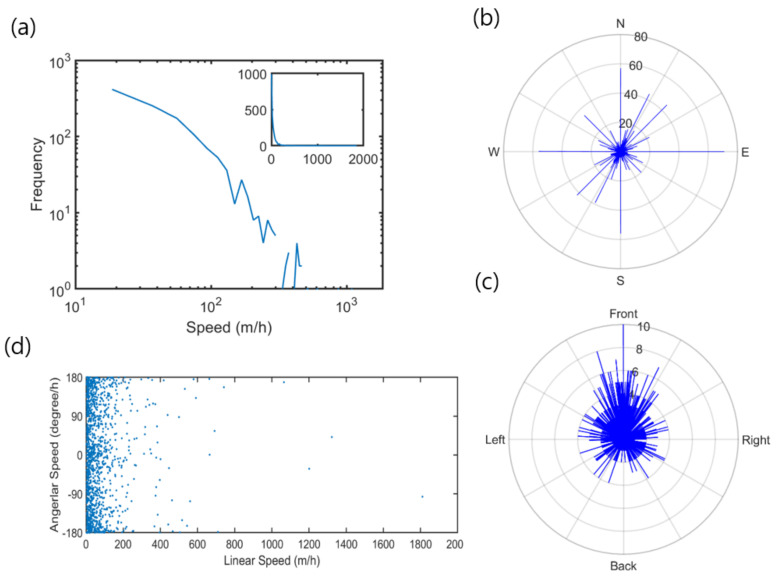
Linear and rotating movement parameters of nutria individuals observed in the Macdo wetland during the survey period; (**a**) frequencies of linear speed in logarithm (inset showing frequencies of linear speed in full scale); (**b**) orientation; (**c**) angular speed; and (**d**) the relation between linear and angular speed.

The majority of the data showed limited activity of nutrias at low speed, intermittently mixed with high-speed movements. The movement average was 48.3 m/h, with a standard deviation of 96.2 m/h. Figure 4a presents the frequency distribution of the linear speed in logarithm (m/h) for all observations during the survey period. Although the speed ranged up to 2000 m/h, most of the observations were below 200 m/h. This result indicates that nutrias generally maintain low activity (see inset in Figure 4a). Based on the movement data, the curve appeared to be initially smooth; the movement decreased rapidly at speed levels below 200 m/h. The long-distance curve was inconclusive, owing to the limited observations.

Figure 4b presents the orientation of nutria movement. Most movements were toward the northeast and southwest along the long axis, which was in accordance with the orientation of the survey area (Figure 1). The angular speed distribution tended to be high when the movement was in the same forward direction (Figure 4c). Figure 4d shows the relationship between linear speed and angular speed. Rotations were more concentrated near 0° or ±180° (i.e., forward direction), within the range of linear speed of 100 m/h.

### 3.2. Spatial Movement Patterns

#### 3.2.1. Nearest-Neighbor Distance According to Sex

**Figure 5:**
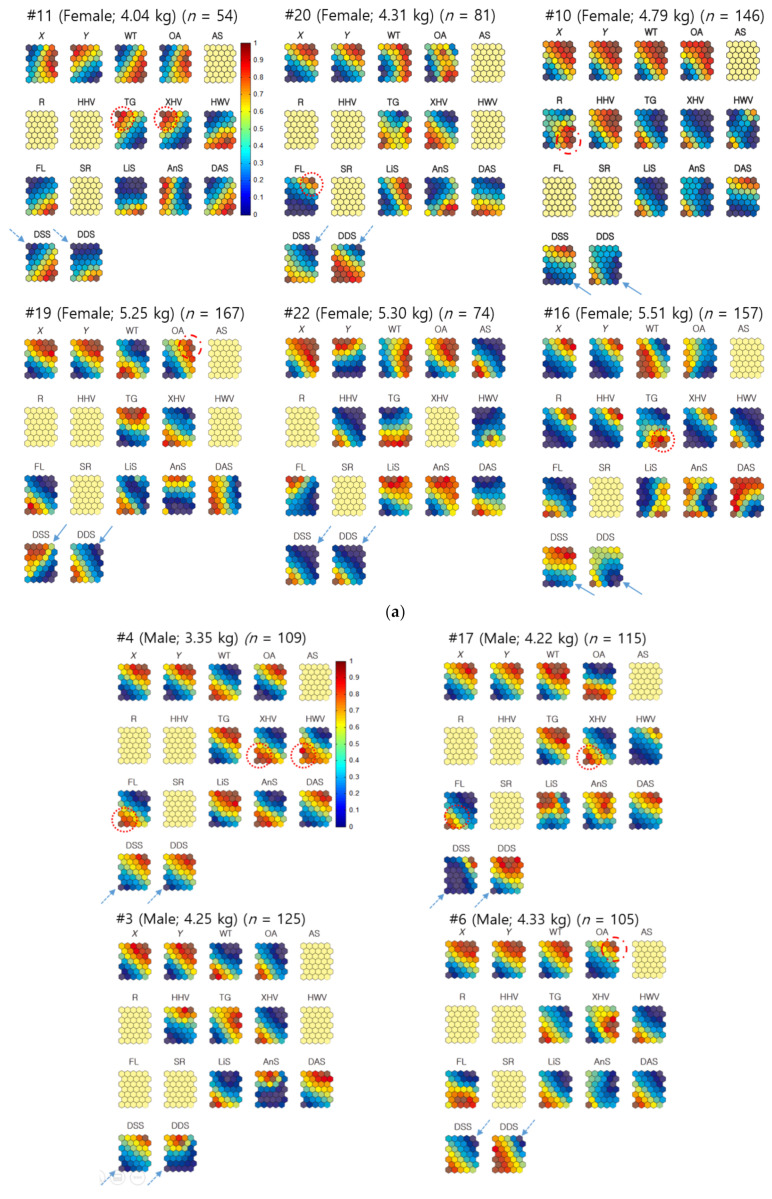

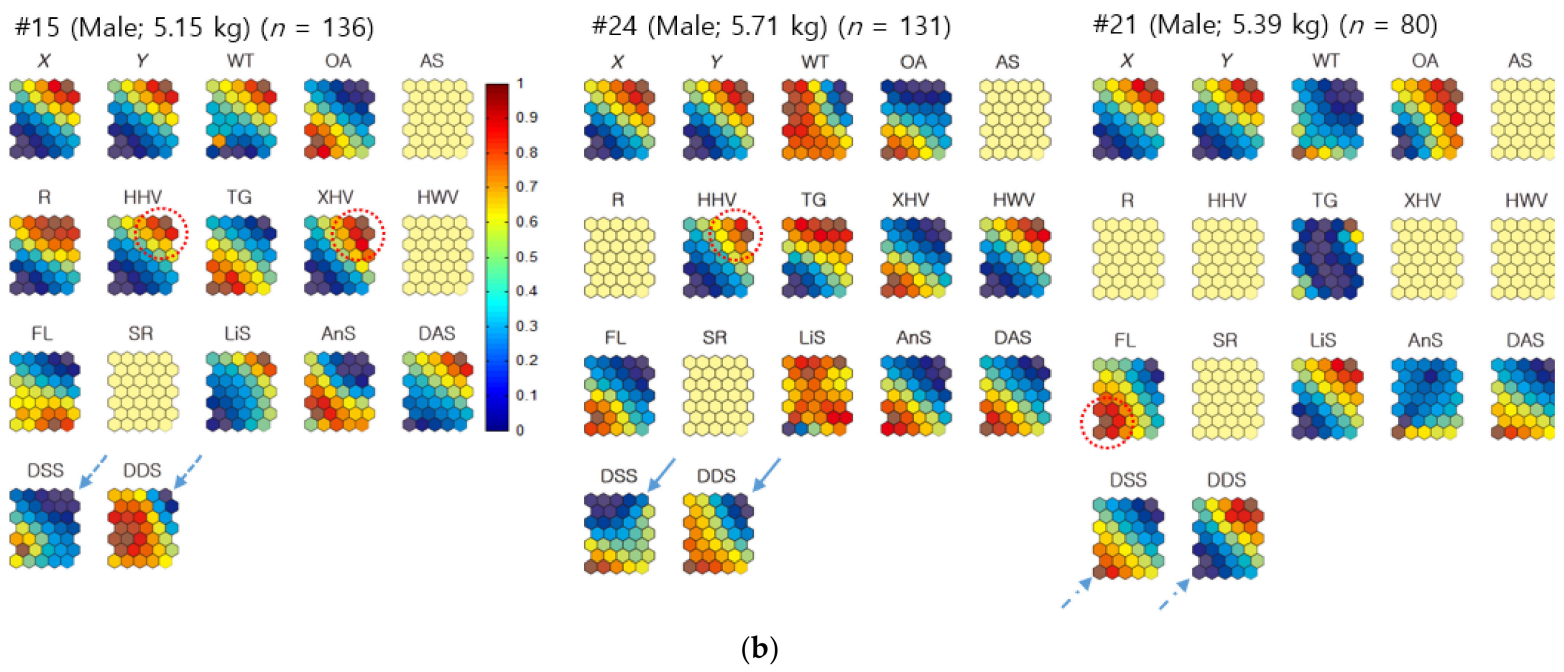
(**a**) Neighbor distances of nutria individuals in association with positions, habitats, plant types, and movement parameters when females were the target individuals for Geo-SOM training (*k* = 3). Values in all the component maps were normalized between 0.0 and 1.0, as shown in the vertical bar. Blank component maps had no input value for the variables in each training. Water temperature: WT; open area: OA; artificial structure: AS; road: R; hydric herbaceous vegetation: HHV; tall grassland: TG; xeric herbaceous vegetation: XHV; hydric woody vegetation: HWV; floating-leaved hydrophytes: FL; sex ratio: SR; linear speed: LiS; angular speed: AnS; distances between all neighbor individuals: DAS; distances between neighbors with the same sex: DSS; and distances between neighbors with different sexes: DDS. (**b**) Neighbor distances of nutria individuals in association with positions, habitats, plant types, and movement parameters when males were the target individuals for Geo-SOM training (*k* = 3). Values in all component maps were normalized between 0.0 and 1.0, as shown in the vertical bar. Blank component maps had no input value for the variables in each training. Water temperature: WT; open area: OA; artificial structure: AS; road: R; hydric herbaceous vegetation: HHV; tall grassland: TG; xeric herbaceous vegetation: XHV; hydric woody vegetation: HWV; floating-leaved hydrophytes: FL; sex ratio: SR; linear speed: LiS; angular speed: AnS; distances between all neighbor individuals: DAS; distances between neighbors with the same sex: DSS; and distances between neighbors with different sexes: DDS.

Since observations were conducted individually, and sample numbers varied according to different individuals, individuals with a sufficient number of samples were selected: this equated to six females (*n* ≥ 54) and seven males (*n* ≥ 80) (Figure 5). The successful convergence in the SOM training of female #11 also allowed this individual to be included in the analysis. Missing data were identified as less than 5% for the distances between neighbors (e.g., male–female [M–F] nearest-neighbor distances). In this case, the average value for each individual was used to replace the missing data for training within the Geo-SOM. The trained data showed movement patterns of nutria individuals on the component planes, in association with variables of environmental factors (water temperature), plant types (e.g., xeric herbaceous vegetation [XHV]), land cover states (e.g., OA), and biological data (e.g., the distance between individuals), using the same and the opposite sex (Figure 5). The ranges of the variables for training with Geo-SOM are presented in Appendix B.

When females were the target individuals for training, the minimum level of nearest-neighbor distances between the different sexes (DDS; female–male [F-M]) matched the minimum level of nearest-neighbor distances between the same sex (DSS; female–female [F-F]) in a majority. For example, female #22 had a minimum DDS (F-M), similar to the minimum DSS (F-F). For #11, and #20, a similar trend was observed (dotted arrows, Figure 5a). For other individuals, such as #10, #16, and #19, the minimum DDS tended to match the intermediate low levels of neighbor distances. However, the profiles were not exact (solid arrows, Figure 5a).

When males were the target individuals, the distances between the minimum DDS (M-F) were also aligned with the minimum DSS male–male [M-M]), including #3, #4, #6, #15, and #17 (dotted arrows, Figure 5b). For male #24, the minimum distance matched the intermediate low level of distance between the same sex on the component plane (solid arrows, Figure 5b). In male #21, however, the minimum DDS (M-F) was inversely associated with the maximum DSS (M-M) (dashed arrows, Figure 5b). These results indicate that the minimum distances are generally in accord between same and different sexes, with occasional contrasting cases.

#### 3.2.2. Neighbor Distances Associated with Biological and Environmental Factors

**Figure 6:**
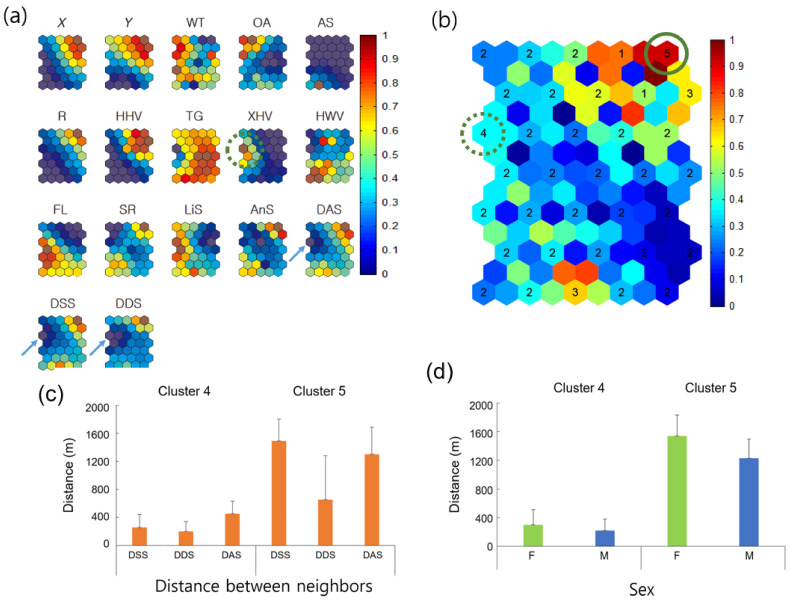
Association of covariates and comparison of neighbor distances for all individuals according to Geo-SOM (*k* = 3). (**a**) neighbor distances in association with environmental and biological factors on the component map (*k* = 3), (**b**) clustering, (**c**) nearest distances between different and same sex, and (**d**) between either females or males. Values in all component maps were normalized between 0.0 and 1.0, as shown in the vertical bar. Water temperature: WT; open area: OA; artificial structure: AS; road: R; hydric herbaceous vegetation: HHV; tall grassland: TG; xeric herbaceous vegetation: XHV; hydric woody vegetation: HWV; floating-leaved hydrophytes: FL; sex ratio: SR; linear speed: LiS; angular speed: AnS; distances between all neighbor individuals: DAS; distances be-tween neighbors with the same sex: DSS; and distances between neighbors with different sexes: DDS. Cluster 4 indicates the minimum distances between neighbors, whereas Cluster 5 provides the maximum distances according to the Geo-SOM training. Mean ± standard deviations are 255.6 ± 186.3 m, 200.5 ± 140.1 m, and 451.8 ± 179.9 m for Cluster 4 (*n* = 381), and 1493.6 ± 309.8 m, 653.7 ± 630.0 m, and 1303.6 ± 383.1 m for Cluster 5 (*n* = 219) for DSS, DDS, and DAS, respectively, in (**c**). Mean ± standard deviations are 299.9 ± 210.4 m (*n* = 169) and 220.4 ± 155.7 m (*n* = 212) for Cluster 4, and 1538.9 ± 293.8 m and 1228.8 ± 265.0 m for Cluster 5 for F and M, respectively, in (**d**).

Figure 5 demonstrates the associations between minimum neighbor distances and plant types and shows that there were variations between individuals. Using females as the target individuals, the minimum DDS (F-M) were associated with TG (#11 and #16), floating-leaved hydrophytes (FL; #20), and xeric herbaceous vegetation (XHV; #11) (dotted circles, Figure 6a). Using males as target individuals (Figure 5b), there were closer associations between minimum DDS (M-F) and plant types. Strong associations were observed with XHV (#4, #15, and #17) and FL (#4, #17, and #21), followed by HHV (#15 and #24), and hydric woody vegetation (HWV; #4) (dotted circles; Figure 5b). The minimum DSS revealed associations with plant types similar to the different sex results. The minimum distances between females related to TG and XHV for individual #11, and FL for individual #20 (Figure 5a). The minimum distances between males were also similarly associated with plant types, including XHV (#4 and #15), followed by HHV (#15), FL (#4), and HWV (#4) (Figure 5b).

The landcover states, OA and road (R), had limited minimum distances for females and males. Minimum DDS were associated with OA for female #19 and R for female #10 (dashed circles, Figure 5a), while the association with land cover state was not clearly found with the minimum DSS for females. For males, DDS and DSS were related to OA in male #6 (dashed circle, Figure 5b).

The minimum DDS and DSS tended to be associated with either high or low temperature without consistency (Figure 5). The associations with geographic locations regarding minimum DDS and DSS values were observed at various places, including the northeast or southwest areas for females (Figure 5a). When males were used as the target individuals, in both DDS and DSS, the nearest-neighbor distances tended to match either the northeast or southwest areas more strongly (Figure 5b).

Figure 6 shows the associations among neighbor distances, environment factors, and movement parameters for all individuals, according to Geo-SOM (*k* = 3). The minimum level of nearest-neighbor distances was consistent for minimum DDS, between the same sexes (DSS), and between all individuals (including both female and male; DAS) on the component map (solid arrows, Figure 6a). The nearest-neighbor distance area matched the XHV plant type (dotted circle, Figure 6a). The areas could be matched to the component maps of location (X, Y) and temperature (WT) on the same Figure 6a, and were low for *X* (west), intermediate for *Y* (further toward the south) for location, and intermediately high for WT regarding temperature. Sex ratio (SR) was defined as the proportion of females and males (1.0 for female and −1.0 for male) in this study. SR is the average of all females and males belonging to a clustered group. If the group consisted of more females than males, the average value would be positive (>0 < 1.0), whereas the value would be negative if more males belonged to the group. Note that values in all the component maps were normalized between 0 and 1.0 for training (vertical bar, Figure 6). The SR value of 0.5 indicates an equal proportion of females and males (Figure 6). SR was in the lower range, corresponding to minimum neighbor distances on the component maps, indicating a higher proportion of males.

Distances between individuals were further checked in the clusters corresponding to maximum and minimum distances trained by Geo-SOM (Figure 6b). Five clusters were formed, according to the *k*-means clustering method (see Materials and Methods) to reveal neighbor distances. Cluster 5, located at the top right corner of both Figure 6b (solid circle) and subplot DDS of Figure 6a, presents the group of maximum distances, whereas Cluster 4, located in the middle of the left edge of both Figure 6b (dotted circle) and subplot DDS of Figure 6a, indicates the group of minimum distances. Figure 6c shows distances in the clusters, including nearest-neighbor DSS, DDS), and DAS. Distances were substantially long (653.7–1493.6 m) in the maximum-distance Cluster 5, whereas distances were short (200.5–451.8 m) in the minimum-distance Cluster 4 (Figure 6c). Distances between the same and different sexes were notable in the maximum-distance Cluster 5; distances between the same sexes were longer (1493.6 m) than distances between different sexes (653.7 m). Besides “distances between DSS and DDS” (*p* = 0.216) in Cluster 4, all other clustered distances in Figure 6c were statistically different (*p* < 0.001), according to the multiple comparison test [58].

Figure 6d further shows the nearest distances between females and males in the maximum- and minimum-distance clusters. The F-F distances were longer than M-M distances, in both minimum distances (299.9 m vs. 220.4 m in Cluster 4) and maximum distances (1538.9 m vs. 1228.8 m in Cluster 5). Statistical significance was observed among all distances (*p* < 0.005 between females and males within the minimum-distance cluster and *p* < 0.001, between all other distances) [58].

**Figure 7:**
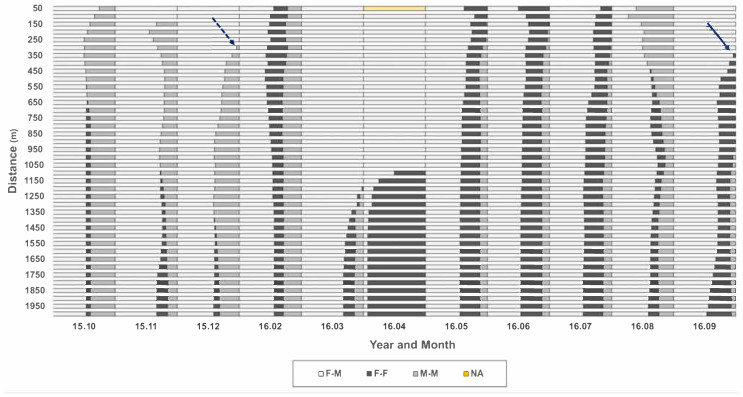
Relative frequencies of co-occurrence of same and different sex individuals across distances from 50 m to 2000 m in the Macdo wetland during the survey period (October 2015–September 2016).

To determine the distance for observing the local co-occurrence of neighbor individuals, all distances from each individual to nearest neighbors were checked across 50 m to 2000 m in a radius from the individual position (*Y*-axis, Figure 7), according to different months of the survey period (*X*-axis, Figure 7). A similar trend in co-occurrence was observed in the short-range up to 250 m. At 300 m, M-M co-occurrence appeared newly in December 2015 (dotted arrow, Figure 7) and increased continuously afterward. At 350 m, F-F co-occurrence was newly observed in September 2016 (solid arrow, Figure 7), increasing afterward continuously. In March and April 2016, co-occurrences of the same sex (F-F and M-M) were found in broad ranges beyond 1100 m. Considering the co-occurrence pattern persisted until 250 m and changed afterward, 250 m was used as the distance for further determining the local co-occurrence of individuals in this study.

**Figure 8:**
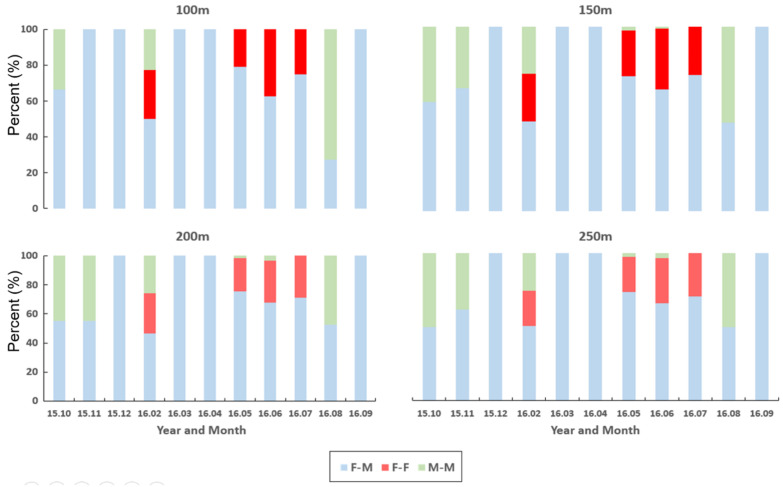
Relative frequencies of co-occurring individuals for the same and different sexes across distances from 100 m to 250 m in the Macdo wetland during the survey period (October 2015–September 2016).

Figure 8 illustrates the relative frequencies of co-occurring individuals within 100, 150, 200, and 250 m distances in different months during the survey period. Seasonal differences were observed in the co-occurrence patterns of individuals. F-M co-occurrences were found briefly in December 2015 (winter) and the following spring (March and April 2016). This result was consistent across different distances. At 100 m, only F-M co-occurrences were observed in November 2015, whereas both F-M and M-M co-occurrences were observed in other distances. An M-M co-occurrence was observed initially in October 2015 (autumn). Then briefly in February 2016 (winter) and again in August 2016 (summer). In contrast, the F-F co-occurrences were observed briefly in late winter (February 2016), followed by early summer (May, June, and July 2016). It is noteworthy that all three types of co-occurrences (F-M, F-F, and M-M) were observed briefly together in February 2015. This trend was overall stable across all distances, and the results show seasonality in local co-occurrences of nutria individuals according to sex.

**Figure 9:**
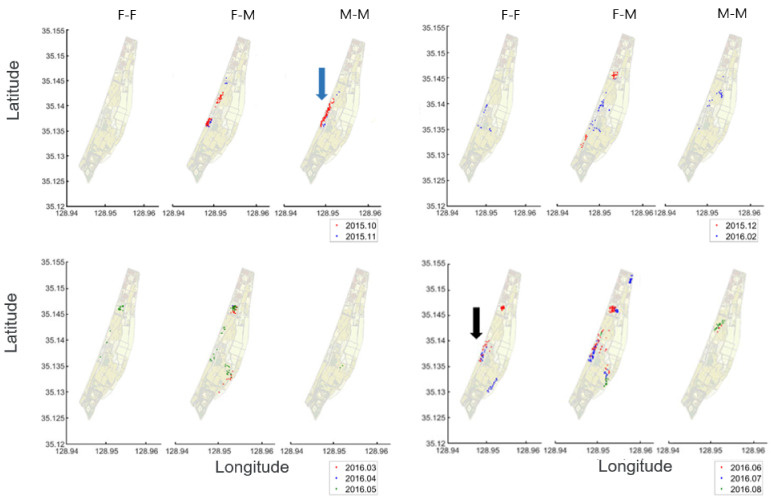
Locations of two individual co-occurrences within 250 m distance in the Macdo wetland during the survey period (October 2015–September 2016).

Figure 9 shows the geographical locations of two individual co-occurrences of nutrias, within 250 m distances in the Macdo wetland during the survey period. Overall, the co-occurrence of the different sexes (female and male) (middle panel in each subfigure in Figure 9) was more frequent when compared with F-F (left panels in each subfigure in Figure 9) and M-M (right panels in each subfigure in Figure 9). F-F co-occurrences were not observed in autumn (October and November 2015), while M-M co-occurrences were scarcely observed in spring (March, April, and May 2016). It is also noteworthy that M-M co-occurrences were more frequently observed in autumn (blue arrow, Figure 9), while F-F co-occurrences occurred more often in summer (black arrow, Figure 9).

### 3.3. Co-Occurrence Patterns in Association with Habitat Types According to Sex

#### 3.3.1. Individual Co-Occurrence Patterns

**Figure 10:**
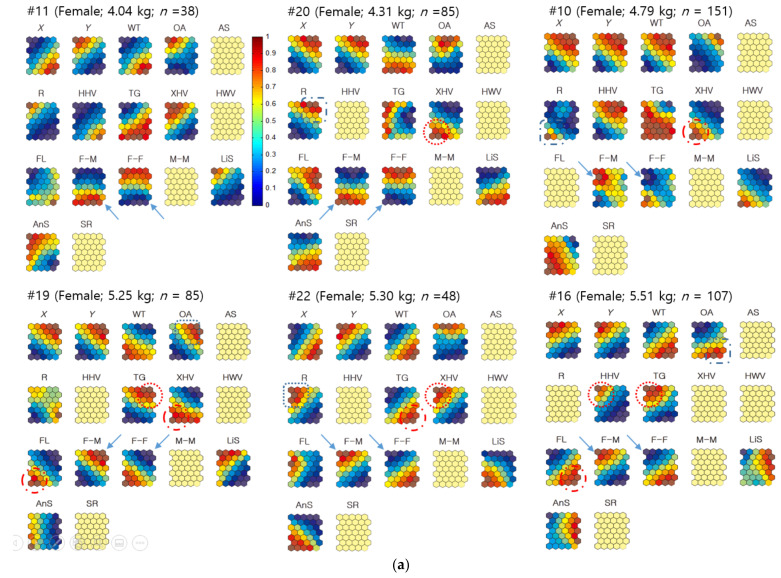

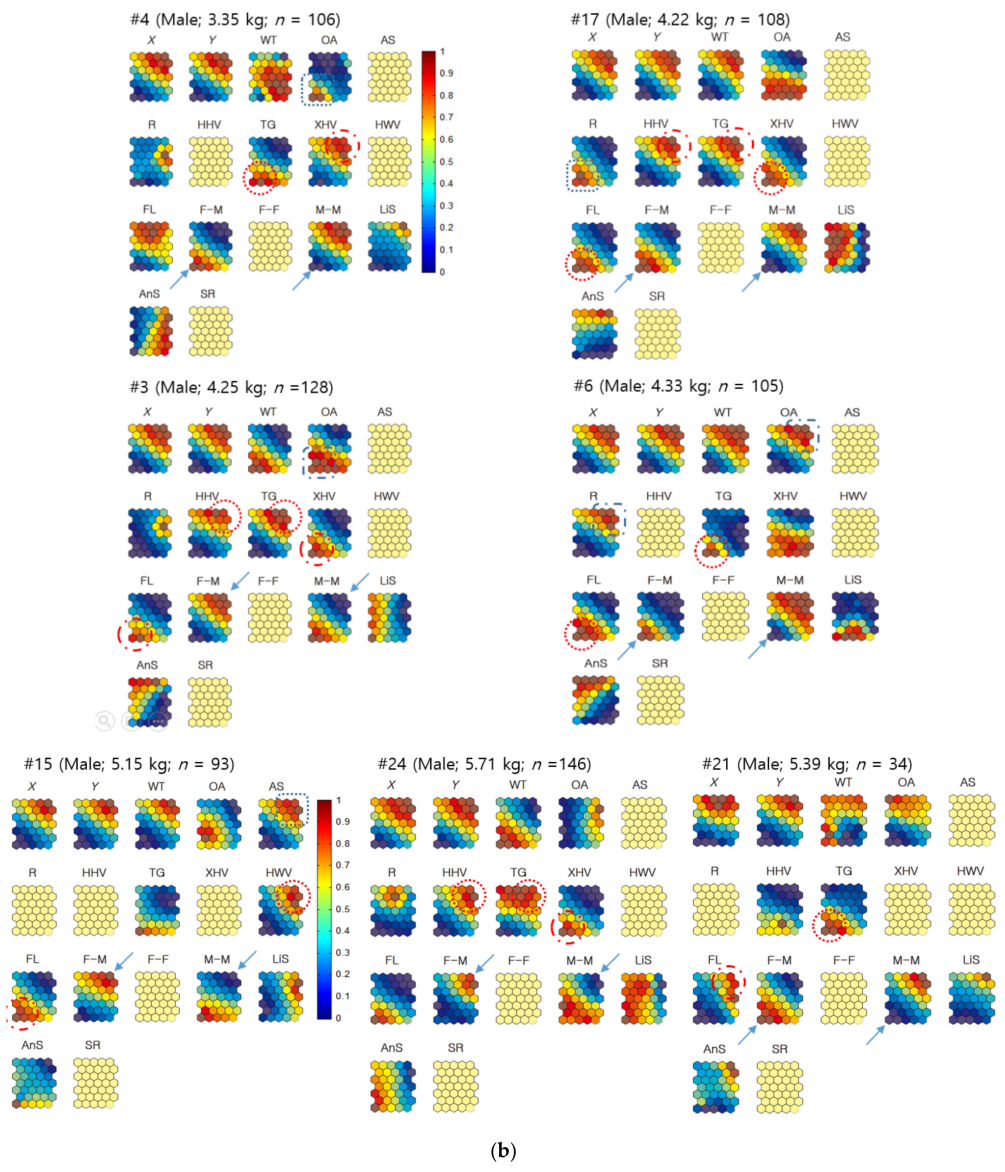
(**a**) Co-occurrence of nutria individuals within the 250 m distance in the Macdo wetland in association with environmental factors and biological data of the females. Values in all component maps were normalized between 0.0 and 1.0, as shown in the vertical bar. Blank component maps had no input value for the variables in each training. Abbreviations include: Water temperature: WT; open area: OA; artificial structure: AS; road: R; hydric herbaceous vegetation: HHV; tall grassland: TG; xeric herbaceous vegetation: XHV; hydric woody vegetation: HWV; floating-leaved hydrophytes: FL; sex ratio: SR; female–male co-occurrence: F-M; female–female co-occurrence: F-F; male–male co-occurrence: M-M; linear speed: LiS; angular speed: AnS. (**b**) Co-occurrence of nutria individuals within the 250 m distance in the Macdo wetland in association with environmental factors and biological data of the males. Values in all component maps were normalized between 0.0 and 1.0, as shown in the vertical bar. Blank component maps had no input value for the variables in each training. Water temperature: WT; open area: OA; artificial structure: AS; road: R; hydric herbaceous vegetation: HHV; tall grassland: TG; xeric herbaceous vegetation: XHV; hydric woody vegetation: HWV; floating-leaved hydrophytes: FL; sex ratio: SR; female–male co-occurrence: F-M; female–female co-occurrence: F-F; male–male co-occurrence: M-M; linear speed: LiS; angular speed: AnS.

Geo-SOM was used to examine how co-occurrences between two individuals within a distance of 250 m, either the same or different sex, were associated with spatial locations and habitat types. Since observations were made individually and sample numbers were variable for different animals, individuals were selected that had enough samples, including six females (*n* ≥ 38) and seven males (*n* ≥ 34). Figure 10a shows component maps of the Geo-SOM (spatial tolerance, *k* = 3) when the training was conducted using females as the target sex. The variable range in the co-occurrence incidence included temperature, plant types, habitat cover states, and biological parameters for training with Geo-SOM, and are presented in Appendix C. It is noteworthy that the maximum incidence of different sexes invariably matched the minimum incidence of the same sex, either for F-F or M-M co-occurrence (solid arrows, Figure 10). For example, female #10 in the analysis using females as target individuals had a maximum incidence of F-M co-occurrence in the top left area of the component map, and was precisely in accordance with the minimum incidence of F-F co-occurrence (solid arrows, Figure 10a). A similar contrary situation was also observed in all other individuals with the minimum incidence of F-M co-occurrences, which was similar to the maximum incidence of F-F co-occurrences (solid arrows, Figure 10a).

When males were the target individuals, the same pattern was observed. The maximum incidence of M-F co-occurrence invariably matched the minimum incidence of M-M co-occurrence for all observed individuals (solid arrows, Figure 10b). The minimum incidence of any M-F co-occurrence was in accordance with the maximum incidence of the M-M co-occurrences, in a reverse manner (Figure 10b), indicating that co-occurrences are opposite between the same and different sexes.

#### 3.3.2. Co-Occurrences Associated with Biological and Environmental Factors

The component maps further demonstrate the co-occurrence of individuals (<250 m) associated with environmental and biological factors according to sex (Figure 10). By matching both global profiles and local compositions on the component map of the Geo-SOM, the levels of association were observed. Overall, co-occurrence patterns were more strongly observed in association with plant types than minimum neighbor distances, as shown in Figure 5. When females were used as the target sex for training, F-M co-occurrences were associated with TG (#16, and #19), XHV (#20 and #22), and HHV (#16) (dotted circles, Figure 10a). Regarding F-F co-occurrences, FL (#16 and #19), XHV (#10 and #19), and TG (#22) were related to the maximum incidence (dashed circles, Figure 10a).

With males as target individuals, M-F co-occurrences were associated with TG (#3, #4, #6, #21, and #24), followed by HHV (#3 and #24), FL (#6 and #17), XHV (#17), and HWV (#15) (dotted circles, Figure 10b). M-M co-occurrences showed that the maximum incidence was associated with FL (#3, #15, and #21), XHV (#3, #4, and #24), HHV (#17), and TG (#17) (dashed circles, Figure 10b).

Table 1 summarizes the associations between co-occurring individuals and plant types. Regardless of the individual, all matched cases were recorded in the table by a visual inspection of the component maps (Figure 10). When more than one association between the co-occurrence and plant type was found for an individual, the total number of associations was listed in the table, whereas no report was included if there was no match. Overall, associations with plant types were higher (11 cases) in M-F co-occurrence (i.e., males used as target individuals) compared with either F-M co-occurrence (i.e., females used as target individuals) or same sex co-occurrences (i.e., F-F (5 cases), M-M (7 cases) (Table 1).

Total TG (7 cases) was mostly associated with co-occurrences between the different sexes, followed by HHV (34 cases) and XHV (34 cases), whereas FL (5 cases) was mostly associated with co-occurrences between the same sex, followed by XHV (4 cases) (Table 1 and Figure 10).

Investigating the land cover states, OA and R had limited co-occurrences (dotted and dashed rectangles, Figure 10). There was a slightly higher association with the OA area for co-occurrences of the same sex. Table 1 lists the co-occurrence associations of individuals with land cover states. Similar to plant types, all cases reported in the table rely on a visual inspection of the SOM component maps, regardless of the individual. A trend of association was observed with OA (6 cases), followed by R (4 cases) (Table 1). No clear patterns were observed in movement parameters. The number of associations was low and variable compared with plant types and land cover states (Figure 10). The association with the location-identified co-occurrences tended to split, mainly into either north-east (NE) or south-west (SW) for data of the same and different sexes (Figure 10). Associations between temperature and co-occurrence were also split into either high or low temperatures. Co-occurrences in different sexes tended to be more associated with low temperature, whereas the associations were reversed for the same sex (Figure 10).

**Figure 11:**
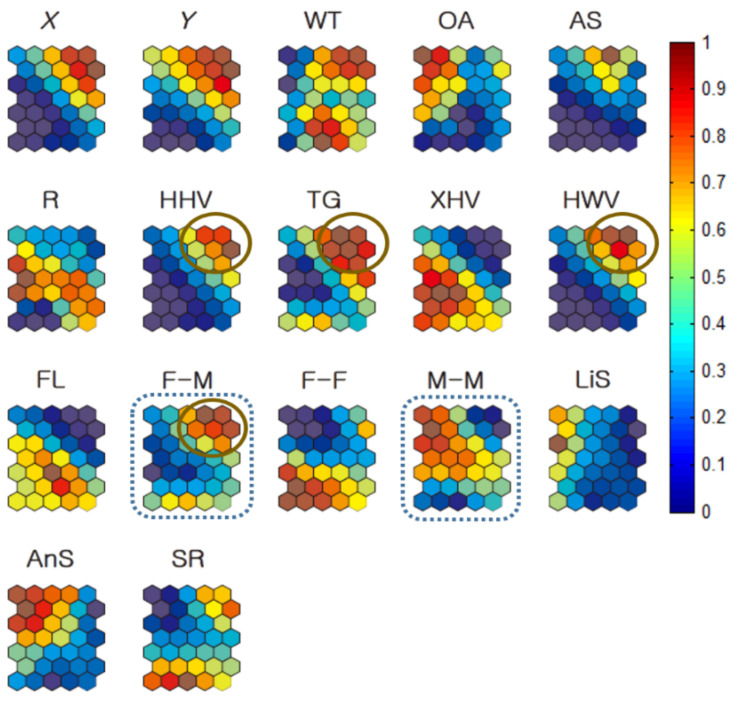
Co-occurrence of nutria individuals within 250 m distances in association with habitat types and locations for all individuals (*k* = 3). All values in the component maps were normalized between 0.0 and 1.0, as shown on the vertical bar. Water temperature: WT; open area: OA; artificial structure: AS; road: R; hydric herbaceous vegetation: HHV; tall grassland: TG; xeric herbaceous vegetation: XHV; hydric woody vegetation: HWV; floating-leaved hydrophytes: FL; sex ratio: SR; female–male co-occurrence: F-M; female–female co-occurrence: F-F; male–male co-occurrence: M-M; linear speed: LiS; angular speed: AnS.

Figure 11 provides the Geo-SOM component planes for the co-occurrence data of all individuals (females and males) trained with the Geo-SOM (*k* = 3). The maximum co-occurrence of F-M inversely matched the minimum incidence of M-M co-occurrences (dotted rectangles, Figure 11). It is noteworthy, however, that the profile of the minimum incidence for F-F was not significantly different to the profile of F-M, when compared with M-M co-occurrence (Figure 11).

Maximum co-occurrences of different sexes overall matched plant types of HHV, HWV, and TG with some variability (solid circles, Figure 11). The maximum M-M incidence (Figure 11) was partly in accordance with lower levels of SR and high levels of OA.

## 4. Discussion

The effective management of alien mammal species that have successfully invaded and established in new environments, requires an in-depth understanding of behavior, habitat preference, and ecological networks [23]. Numerous theoretical and practical studies have been conducted on nutrias, including synecology and behavioral traits [59,60,61,62], feeding patterns [63,64,65], movement [66,67,68,69], and active regions [62,70,71,72,73].

Nutria is an aggregative rodent species, usually forming a group of one adult male, with one or a few adult females and young individuals [60,61]. Nutria groups have a sex ratio of approximately 0.6: 1.6 (male: female) [18]. Nutria populations are well adapted to diverse environments, with a tendency to favor freshwater habitats [69]. Their behavior is influenced by environmental factors (e.g., water temperature and food availability), other organisms (e.g., competitive species, predation), and human impacts (e.g., capture) of the inhabited area [74].

However, specific behavioral information on continuous individual movement has not been available for nutrias. This is partly due to the difficulty of continuously recording individual behavior in the field, as well as difficulties in analyzing complex behavioral data. Information on habitat preferences with movement is crucial in improving understanding of the feeding and resting behaviors of nutrias. Moreover, spatial co-occurrence of different individuals provides necessary information on nutria sociality, including mating, foraging, and territoriality. Understanding spatial movement and inter-individual relationship patterns is essential for effective population management.

This study utilized a machine learning tool, Geo-SOM, to extract the complex data of nutria movement associated with various factors, including spatial positions, environmental factors, and biological data. The results demonstrate that Geo-SOM can effectively illustrate spatial distance and co-occurrence patterns of nutrias from individuals observed continuously in the wild (Figure 5, Figure 6, Figure 7, Figure 8, Figure 9, Figure 10 and Figure 11). It was predicted that males would play a significant role in spacing with other individuals. The negative associations between M-F co-occurrences versus M-M co-occurrences suggest male dominance in determining the co-occurrence of different sexes (Figure 10 and Figure 11). Whereas the areas of the component map using ‘M-F’ and ‘M-M’ were negative, the area for ‘F-F’ was not closely related to either ‘F-M’ or ‘M-M’. This indicates a greater influence of M-M co-occurrence than F-F co-occurrence in determining the spacing of nutria individuals. The prediction regarding male dominance was met, indirectly supporting the theory of the dominant role of males in nutria spacing in the wild. Nutrias are known for their male-oriented group formation; therefore, adult male nutrias aggressively maintain their territory. As a male matures, it becomes independent and leaves the group [61]. Female adults are ready to mate within 1–2 days [75]. Rapid mating availability may be a reason for young males to remain close to the old groups. The close positioning of individuals when males and females co-occur, as stated above, would provide a good opportunity for young males to form their own group.

TG was mostly associated with co-occurrences between the different sexes, followed by HHV and XHV. Meanwhile, FLs were closely related to co-occurrences between the same sexes, followed by XHV (Figure 10, Table 1). Besides XHV, compositions of plant types varied according to co-occurrences of the same and different sexes. In different sexes, TG and HHV may play a role in the behaviors that occur between females and males, for example, mating. Regarding the same sex, XHV (4 cases) was more strongly associated with co-occurrences than HHV (1 case) (Table 1). Considering that co-occurrences in the same sex were mostly associated with FL (5 cases), FL and XHV may be associated with behaviors not directly related to sexual activity, such as feeding. It was conjectured that local co-occurrences of individuals would be associated with environmental conditions. The results support the relationships between co-occurrences and plant types. However, clear relationships between plant types and the nearest distances of nutrias were not conclusive in this study. Future research should unravel the relationship between food sources and the spacing of nutria populations. Regarding land cover states, co-occurrences were observed with a trend of association with OA and R. However, the number of cases was not sufficient, and this habitat relationship also needs further study, with additional observations. It was speculated that the distances between different sexes would be shorter between different sexes than between the same sex, considering the possibility of positive relationships between different sexes (e.g., mating) and negative relationships between the same sex (e.g., competition). Neighbor distances were significantly longer between the same sex than between different sexes, e.g., the maximum-distance group; 1493.6 m and 653.7 m, respectively (Figure 6c). This indicates that a greater distance was required between individuals of the same sex, supporting our prediction. Contradictory to this conjecture, however, distances between females were greater than distances between males. Females had more space in both maximum and minimum-distance groups; 1538.9 m and 299.9 m, respectively. Males had less space between males; 1228.8 m and 220.4 m, respectively (Figure 6d). In this study, the females and males were all adults; therefore, the young individuals who closely followed their mothers were not included. Adult females require more spacing than males, indirectly indicating that females avoid being in contact with each other. One reason is that males may intentionally influence females to locate far from other females. However, it is too early to make any conclusion in this regard, and more studies are required, including dominant/submissive and territorial behavior of nutrias.

The unique behavioral characteristic of spacing between neighboring individuals may be influenced by local environmental (e.g., habitat type) and biological (e.g., movement, presence of neighboring individuals) factors, as stated above. This is essential information for the successful management of nutrias currently being undertaken by the Korean government. Once nutrias invade new ecosystems, the invading populations select life strategies that optimize the new environment. Information on individual movement, in association with environmental and biological factors, is essential for determining the most effective control practices, and can serve as a basis for establishing large-scale management strategies at the national level.

Geo-SOM provides explicit spatial information, allowing the development of space-oriented management policies that can be used to further extending our knowledge of habitat preference in population expansion, as observed with another mammalian species, otter, *Lutra lutra* [76]. Geo-SOM results could be effectively linked with species distribution models (SDMs) regarding habitat suitability and population distribution. SDMs have been used to predict the potential range expansion of nutrias, with a machine learning technique, MaxEnt [31], notably with a hierarchical approach consisting of broad-scale (climate) and local-scale (habitat) models [32]. This study addresses movement behaviors of individuals on a refined scale; therefore, the results could be effectively linked to SDM results to interpret species distribution precisely. For instance, relationships between plant types in habitats, which are variates of SOM, and local co-occurrences of the same and different sexes, which could be variates of SDM, would provide in-depth information on the spatial distribution of nutrias by extrapolating the population distribution to the relations between “population distribution” and “individual behavior”. Alternatively, Geo-SOM results could also be used to define risk factors and provide effective management strategies (e.g., control threshold), which can be adopted to local regions, as observed in aquatic ecosystems [55]. In addition, the current study could be further extended to determine behavior with the use of mathematical models, including the hidden Markov model [77,78]. The study training with spatial data with machine learning can also be extrapolated to define home ranges pertaining to local habitat conditions in the future.

In this study, few samples were recorded because of the difficulty in making continuous telemetry measurements in the wild. Furthermore, the survey only occurred over three days per month (see Materials and Methods). Although individuals with relatively a large number of observations (six females and seven males) were used for training with Geo-SOM, data for a longer period were not available in this study. Therefore, detailed information on the associations with environmental factors and biological parameters could not be collated. The observation times were also recorded, but the time of the day was not included as a covariate for the Geo-SOM training, owing to the small number of samples obtained in this study. Future research should include more surveys over extended periods to monitor movements and habitat preferences in the spatial framework.

## 5. Conclusions

The spatial distribution patterns of nutrias are effectively illustrated, in association with diverse environmental and biological factors, using a machine learning technique, Geo-SOM. Valuable information was obtained regarding the nearest minimum distances and local co-occurrences of neighbors, according to sex. The minimum DDS were, overall, related to the minimum DSS. F-M local co-occurrences were more frequently observed than F-F and M-M co-occurrences and varied with season. Local co-occurrence patterns between females and males were negatively associated with M-M co-occurrences compared with F-F co-occurrence, suggesting a male dominance in determining the co-occurrence of nutria individuals. TG was mostly associated with co-occurrences between different sexes, followed by HHV. Conversely, FL was mainly associated with co-occurrences between the same sexes, followed by herbaceous vegetation. Unique behavioral research investigating movement behavior, based on the information extracted by informatics, is essential for monitoring and managing nutrias. In the future, more detailed research is required to understand behavioral states and identify spatial management strategies for nutria populations.

## Figures and Tables

**Table 1:** Co-occurrence of nutria individuals in association with plant types and land cover states, according to a visual inspection of the Geo-SOM component maps (all observed cases reported regardless of individuals).

Neighbors		Plant Types						Land Cover States			
		TG	FL	XHV	HHV	HWV	Total	OA	R	AS	Total
Different sex	F-M	2	0	2	1	0	5	1	1	0	2
	M-F	5	2	1	2	1	11	1	1	1	3
	Subtotal	7	2	3	3	1	16	2	2	1	5
Same sex	F-F	1	2	2	0	0	5	1	2	0	3
	M-M	1	3	2	1	0	7	2	1	0	3
	Subtotal	2	5	4	1	0	12	3	3	0	6
Total		9	7	7	4	1	28	5	5	1	11

**Figure A1:**
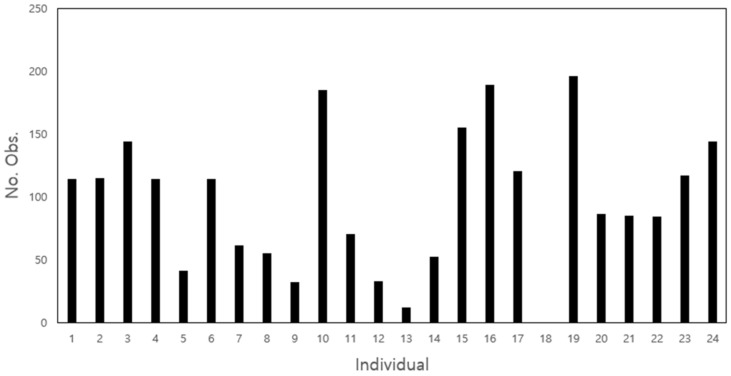
Total number of recordings per individual (**top panel**) and information collected during the survey on the observed individuals (**bottom panel**) in the Macdo wetland.

## Data Availability

All data generated or analyzed during this study are included in this article.

## Appendix A

**Individual**	**No. 1**	**No. 2**	**No. 3**	**No. 4**	**No. 5**	**No. 6**	**No. 7**	**No. 8**	**No. 9**	**No. 10**	**No. 11**	**No. 12**
Sex	Female	Female	Male	Male	Male	Male	Male	Female	Male	Female	Female	Male
Weight (Kg)	6.12	3.13	4.25	3.35	3.73	4.33	6.55	4.35	5.18	4.79	4.04	5.36
Individual	No. 13	No. 14	No. 15	No. 16	No. 17	No. 18	No. 19	No. 20	No. 21	No. 22	No. 23	No. 24
Sex	Female	Female	Male	Female	Male	Male	Female	Female	Male	Female	Female	Male
Weight (Kg)	3.84	5.27	5.15	5.51	4.22	4.74	5.25	4.31	5.39	5.30	5.20	5.71

## Appendix B

Range of each variable: nearest-neighbor distances, temperature, plant types, habitat cover state, and biological parameters used with Geo-SOM.

**Indivdua**	***n***	**Sex**	**Range**	***X***	***Y***	**WT**	**OA**	**AS**	**R**	**HHV**	**TG**	**XHV**	**HWV**	**FL**	**SR**	**LiS**	**AnS**	**DAS**	**DSS**	**DDS**
11	54	Female	Min	128.9459	35.1245	4.4	0	0	0	0	0	0	0	0	1	0.6	0	208.2	21.2	24.8
			Max	128.9525	35.1394	16.2	1	0	0	0	1	1	1	1	1	928.4	234.8	1859.6	2227.1	1905.9
20	81	Female	Min	128.9476	35.1358	20.7	0	0	0	0	0	0	0	0	1	0.3	0	297.8	14.2	10
			Max	128.9516	35.1418	26.9	1	0	0	0	1	1	0	1	1	286.6	292.2	1117.9	933	761.3
10	146	Female	Min	128.9469	35.1336	4.4	0	0	0	0	0	0	0	0	1	0.1	0	36.8	7.9	8.8
			Max	128.9555	35.1468	26.9	1	0	1	1	1	1	1	0	1	472.6	420.3	2102.1	2227.1	1819.3
19	167	Female	Min	128.9479	35.1357	20.7	0	0	0	0	0	0	0	0	1	0.1	0	148.6	14.2	11.5
			Max	128.9522	35.1432	30.9	1	0	0	0	1	1	0	1	1	378.9	430.1	1487.3	1843.2	847
22	74	Female	Min	128.9436	35.1275	20.7	0	0	0	0	0	0	0	0	1	0.5	0.1	47.5	23.6	90.7
			Max	128.9524	35.139	26.9	1	1	0	1	1	0	1	1	1	315.8	289.3	2217.6	2102.1	2088.2
16	157	Female	Min	128.9497	35.1296	20.7	0	0	0	0	0	0	0	0	1	0.1	0	47.5	47.5	8.8
			Max	128.9581	35.1526	30.9	1	0	1	1	1	1	1	1	1	168.4	355.4	1434.7	2092.5	964.4
4	109	Male	Min	128.9475	35.135	10	0	0	0	0	0	0	0	0	−1	0.4	0	207.6	8.8	15
			Max	128.9523	35.1432	20.8	1	0	0	0	1	1	1	1	−1	713.9	523.3	1537.8	2868.4	1207.6
17	115	Male	Min	128.9477	35.1358	20.7	0	0	0	0	0	0	0	0	−1	0.2	0	105.2	27.4	11.5
			Max	128.9542	35.1461	30.9	1	0	0	0	1	1	1	1	−1	245.4	383	1157.8	1223.9	1091.7
3	125	Male	Min	128.938	35.1171	4.4	0	0	0	0	0	0	0	0	−1	0.2	0	52.7	16.2	8.8
			Max	128.9545	35.1467	20.8	1	0	0	1	1	1	0	0	−1	461.2	659.9	2594.9	2868.4	1637.7
6	105	Male	Min	128.9458	35.1316	10	0	0	0	0	0	0	0	0	−1	0.3	0	193	8.8	37.1
			Max	128.9528	35.1447	20.8	1	0	0	0	1	1	1	1	−1	443.4	277.4	1140.8	1065.2	1377
15	136	Male	Min	128.9497	35.1296	20.7	0	0	0	0	0	0	0	0	−1	0.3	0	93.2	127.9	3.9
			Max	128.9582	35.153	30.9	1	0	1	1	1	1	0	1	−1	225.6	321.1	1548.8	1404.5	1208.4
24	131	Male	Min	128.9476	35.1358	20.7	0	0	0	0	0	0	0	0	−1	0	0	50.5	27.4	3.9
			Max	128.9545	35.1467	30.9	1	0	0	1	1	1	1	1	−1	199.6	365.4	1200.6	1316.5	1200.6
21	80	Male	Min	128.9508	35.1338	20.7	0	0	0	0	0	0	0	0	−1	0.6	0.1	223.6	108.5	90.7
			Max	128.9537	35.1431	26.9	1	0	0	0	1	0	0	1	−1	174.8	266.6	1084.7	1126.4	828.6

## Appendix C

Range of each variable including co-occurrence incidence, temperature, plant type, habitat cover state, and biological parameters used with Geo-SOM.

**Individual**	***n***	**Sex**	**Range**	***X***	***Y***	**WT**	**OA**	**AS**	**R**	**HHV**	**TG**	**XHV**	**HWV**	**FL**	**F-M**	**F-F**	**M-M**	**LiS**	**AnS**	**SR**
11	38	Female	Min	128.95	35.13	4.4	0	0	0	0	0	0	0	0	0	0	0	0.6	1.3	1
			Max	128.95	35.14	10.7	1	0	1	1	1	1	0	1	1	1	0	928.4	180	1
20	85	Female	Min	128.95	35.14	20.7	0	0	0	0	0	0	0	0	0	0	0	1.7	0.2	1
			Max	128.95	35.14	26.9	1	0	1	0	1	1	0	1	1	1	0	286.6	180	1
10	151	Female	Min	128.95	35.13	4.4	0	0	0	0	0	0	0	0	0	0	0	0.6	0.2	1
			Max	128.95	35.15	26.9	1	0	1	1	1	1	0	0	1	1	0	472.6	180	1
19	85	Female	Min	128.95	35.14	20.7	0	0	0	0	0	0	0	0	0	0	0	0.8	0.5	1
			Max	128.95	35.14	30.9	1	0	1	0	1	1	0	1	1	1	0	378.9	180	1
22	48	Female	Min	128.95	35.13	24.7	0	0	0	0	0	0	0	0	0	0	0	0.6	1.1	1
			Max	128.95	35.14	26.9	1	0	1	0	1	1	0	1	1	1	0	315.8	180	1
16	107	Female	Min	128.95	35.13	20.7	0	0	0	0	0	0	0	0	0	0	0	0.4	1.2	1
			Max	128.95	35.14	30.9	1	0	0	1	1	0	0	1	1	1	0	140.1	180	1
4	106	Male	Min	128.95	35.14	15.7	0	0	0	0	0	0	0	0	0	0	0	1.7	3	−1
			Max	128.95	35.14	20.8	1	0	1	0	1	1	0	1	1	0	1	713.9	180	−1
17	108	Male	Min	128.95	35.14	20.7	0	0	0	0	0	0	0	0	0	0	0	1.1	1.8	−1
			Max	128.95	35.15	30.9	1	0	1	1	1	1	0	1	1	0	1	245.4	180	−1
3	128	Male	Min	128.95	35.14	4.4	0	0	0	0	0	0	0	0	0	0	0	0.7	0	−1
			Max	128.95	35.15	20.8	1	0	1	1	1	1	0	1	1	0	1	461.2	180	−1
6	105	Male	Min	128.95	35.13	10	0	0	0	0	0	0	0	0	0	0	0	1.3	0	−1
			Max	128.95	35.15	20.8	1	0	1	0	1	1	0	1	1	0	1	191.5	180	−1
15	93	Male	Min	128.95	35.13	20.7	0	0	0	0	0	0	0	0	0	0	0	0.8	0	−1
			Max	128.96	35.15	30.9	1	1	0	0	1	0	1	1	1	0	1	225.6	180	−1
24	146	Male	Min	128.95	35.14	20.7	0	0	0	0	0	0	0	0	0	0	0	1	0.7	−1
			Max	128.95	35.15	30.9	1	0	1	1	1	1	0	1	1	0	1	199.6	180	−1
21	34	Male	Min	128.95	35.13	20.7	0	0	0	0	0	0	0	0	0	0	0	2.5	0.1	−1
			Max	128.95	35.14	26.9	1	0	0	1	1	0	0	1	1	0	1	161.4	180	−1
